# Detection and identification of the phytoplasma associated with China ixeris (*Ixeridium chinense*) fasciation

**DOI:** 10.1186/1999-3110-54-52

**Published:** 2013-10-31

**Authors:** Zheng-Nan Li, Ping Liu, Lei Zhang, Yun-Feng Wu

**Affiliations:** 1 State Key Laboratory of Crop Stress Biology for Arid Areas/Key Laboratory of Integrated Pest Management on Crop in Northwestern Loess Plateau, Ministry of Agriculture/Key Laboratory of Plant Protection Resources and Pest Management, Ministry of Education, College of Plant Protection, Northwest A&F University, Yangling, 712100 Shaanxi Province P. R. China; 2 College of Forestry, Northwest A&F University, Yangling, Shaanxi 712100 P. R. China

**Keywords:** Transmission electron microscopy, 16S rRNA and *tuf* genes, PCR, Phylogeny analysis, RFLP

## Abstract

**Background:**

Phytoplasmas are always associated with symptoms in host plants such as stunting of stems, witches’-broom, yellowing of leaves, formation of sterile-deformed flowers, virescence and phyllody. Recently also symptom of fasciation was reported associated with phytoplasma presence. In the present work, China ixeris fasciation was observed associated with phytoplasmas in Guanzhong Area, Shaanxi, China.

**Results:**

Phytoplasma-like bodies were observed under transmission electron microscope in stem tissues of symptomatic samples. The 16S rRNA operon and *tuf* genes from phytoplasmas were amplified by PCR assays. Phylogenetic trees were calculated respectively based on sequences data of these two genes. The pattern of restriction fragment length polymorphism (RFLP) was generated via digesting the PCR products of 16S rRNA gene with eight restriction enzymes.

**Conclusion:**

The presence of phytoplasma in China ixeris exhibiting fasciation symptom was confirmed by the results of TEM observation and PCR testing. Based on sequence data, phylogeny analysis and actual restriction fragment length polymorphism (RFLP) analysis, the associated phytoplasma was classified as related to 16SrI-C subgroup. This was the first record of phytoplasmas in China ixeris.

**Electronic supplementary material:**

The online version of this article (doi:10.1186/1999-3110-54-52) contains supplementary material, which is available to authorized users.

## Background

Fasciation is a result of an aberrant growth development in plants. A fasciated plant often forms flattened, wider than normal stems similar to a multiple stem fusion. This abnormal phenotype is observable in many plant species in nature, and spontaneous genetic mutations, bacterial infections, insect or mite attacks, physically wounding or herbicide sprays were reported causing plant fasciations (Goethals et al., 2001). More recently, phytoplasma were detected in some plants exhibiting fasciation symptom (Bertaccini et al., 2005; Poncarová‒Vorácková et al., 1998; Fránová and Petrzik, 2010; Kumar et al., 2010; Wu et al., 2012; Li et al., 2012).

Phytoplasmas were assigned to a provisional genus, ‘*Candidatus* Phytoplasma’ within the class *Mollicutes* (IRPCM, 2004). They are cell-wall-less prokaryotes that inhabit plant phloem system, associated with symptoms such as stunting of stems, proliferation of axillary shoots (witches’-broom), yellowing of leaves, formation of sterile-deformed flowers, greening of floral tissues (virescence) and forming of leaf-like flower organs (phyllody). Since phytoplasmas have just been cultured under axenic conditions recently (Contaldo et al., 2012), their diagnosis still depends on molecular analysis of several evolutionarily conserved genes like 16S ribosomal RNA (rRNA), ribosomal protein (*rp*) and elongation factor TU (*tuf*). So far, a total of 31 phytoplasma groups and over 50 subgroups were found based on analysis of 16S rRNA gene sequences (Wei et al., 2007; Zhao et al., 2009; Lee et al., 2011).

China ixeris [*Ixeridium chinense* (Thunb.) Tzvel.] is a perennial herb of the family *Compositae*, which could be used as traditional Chinese herbal medicine for treatment of enteritis and cholecystitis (Liu et al. 2010). In this report, China ixeris fasciation disease occurrence is described and a phytoplasma related to 16SrI-C subgroup was associated with the symptomatology described.

## Methods

### Sample collection

In May 2009, China ixeris fasciation was observed in Guanzhong Area, Shaanxi Province, China. To verify disease aetiology, 30 symptomatic and three asymptomatic plants were collected in field. Some China ixeris plants were also transplanted into an insect-proof greenhouse for observation of symptom development.

### Transmission electron microscopy

Fasciated stems of ca. 2 × 2 mm^2^ from symptomatic samples were processed for transmission electron microscope examination. The tissues were fixed in a buffer (pH 7.2) containing 3% (v/v) glutaraldehyde and 4% (v/v) paraformaldehyde, incubated at 4°C for 4 h, and subsequently in 1% (v/v) osmium tetroxide at room temperature for 2 h. Then the fixed samples were dehydrated in concentration gradients of ethanol (10-70%) and acetone (0-100%), and finally were embedded in Epon 812 Kamińska et al., (2001). The ultra-thin sections were stained with uranyl acetate and led citrate, and then examined.

### PCR amplification

Total DNA of each sample was extracted following cetyltrimethyl ammonium bromide (CTAB) method (Kollar et al., 1990), and used as template in PCR assays. Total DNA of samples infected with a phytoplasma related to wheat blue dwarf phytoplasma (WBDp) (16SrI-C subgroup) (Wu et al., 2010) and chinaberry witches’ broom phytoplasma (CWBp) (16SrI-B subgroup) (Wu et al., 2010) were as positive controls.

Primers P1 (Deng and Hiruki, 1991) and P7 (Schneider et al., 1995) were used in amplification of phytoplasma 16S rRNA gene, spacer region between 16S and 23S rRNA genes and the start of 23S rRNA gene. The PCR products were diluted 1: 29 with sterile double-distilled water prior to the nested amplification using the general primer pair R16F2n/R2 (Gundersen and Lee, 1996). Each PCR mixture (25 μL) contained: 2 μL DNA template (10 ng/uL), 1 μL (10 pM) of each primer, 2 μL dNTP (2.5 mM), 2 μL MgCl_2_ (25 mM), 2.5 μL 10 × Taq buffer and 1 U *Taq* DNA polymerase (Thermo Fisher Scientific Inc.), and sterile double-distilled water to the final volume. The PCR amplification program was as follows: preheating at 94°C for 3 min, and then subjected them to 35 amplification cycles, of denaturation at 94°C for 1 min, annealing at 50°C for 1 min, and extension at 72°C for 1 min, with a final elongation of 72°C for 10 min.

Amplification of phytoplasma *tuf* gene was primed by primer pair fTufu/rTufu (Schneider and Gibb, 1997). The reaction mixture was set as above. After 3 minutes’ preheating at 94°C, 30 amplification cycles were carried out: denaturation at 94°C for 30 s, annealing at 50°C for 30 s and extension at 72°C for 1 min, with a final elongation of 72°C for 10 min.

PCR products were separated in 1% agarose gel by electrophoresis, stained with ethidium bromide and visualized using UV transilluminator.

### Cloning, sequencing and sequence analysis

PCR products of 1.8 kb (phytoplasma 16S rRNA gene) and 0.8 kb (*tuf* gene) were purified using a commercial PCR Purification Kit (Bio Teke Corporation, Beijing, China) and cloned. The clones contained recombinant plasmid were selected by blue-white screen and for each sample, three clones were selected and sequenced by TaKaRa Biotechnology (Dalian) Co., Ltd. The primers used for sequencing of 16S rRNA gene were M13F(-47): CGCCAGGGTTTTCCCAGTCACGAC/M13R(-48): AGCGGATAACAATTTCACACAGGA and of *tuf* gene were M13F(-77): GATGTGCTGCAAGGCGATTA/M13R(-48), which were designed based on the sequence of pMD18-T simple vector and offered by the TaKaRa Biotehnology Co. Ltd.

Sequences were aligned using the Lasergene software (version 7.0; DNASTAR, Madison, USA) and used for searching against the database of National Center for Biotechnology Information (NCBI) by BLASTn.

Published phytoplasma sequences were retrived from GenBank; 33 sequences of 16S rRNA gene and 17 sequences of *tuf* genes from groups 16SrI, -III, -V, -X and -XII were selected. Phylogenetic trees were built by neighbor-joining (16S rRNA) or maximum parsimony (*tuf* gene) methods with a 1000-replicate bootstrap search using MEGA4 (Saitou and Nei, 1987; Tamura et al., 2007).

The nested PCR products of 16S rRNA gene (1.2 kb) from symptomatic samples were concentrated, and digested with eight restriction enzymes *Alu* I, *Bfa* I, *Hae* III, *Hha* I, *Hpa* II, *Kpn* I, *Mse* I and *Rsa* I (Lee et al., 1998). The digested PCR products were separated in 8% polyacrylamide gel by electrophoresis and visualized using UV tranilluminator after ethidium bromide staining.

## Results

**Figure 1:**
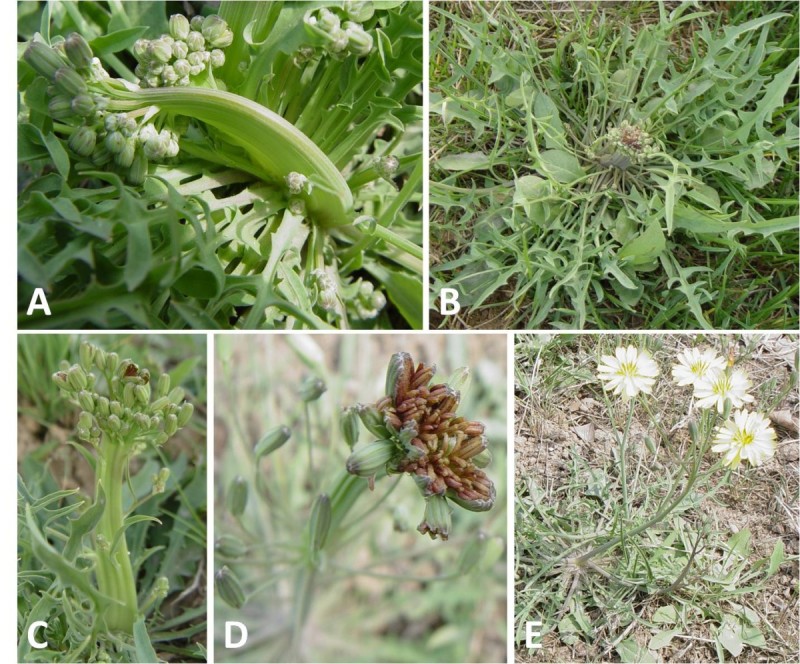
**Symptoms of phytoplasma-infected China ixeris.** The symptoms are curving stem **(A)**; flat stem, shorting stalk **(C)**; narrowing leaves **(B)** and clustering of multi-inflorescence **(D)**, compared to healthy one **(E)**.

In field, the phytoplasma-infected China ixerises exhibited symptoms of curving and flat stem (Figure 1A, 1C), narrow leaves (Figure 1B) and clustering of multi-inflorescence (Figure 1D). The disease was named China Ixeris fasciation (ChIF). After two months’ observation of field-collected China Ixerises for symptom development in green house, the flat stem did not broaden and the abnormal flowers did not seed.

**Figure 2:**
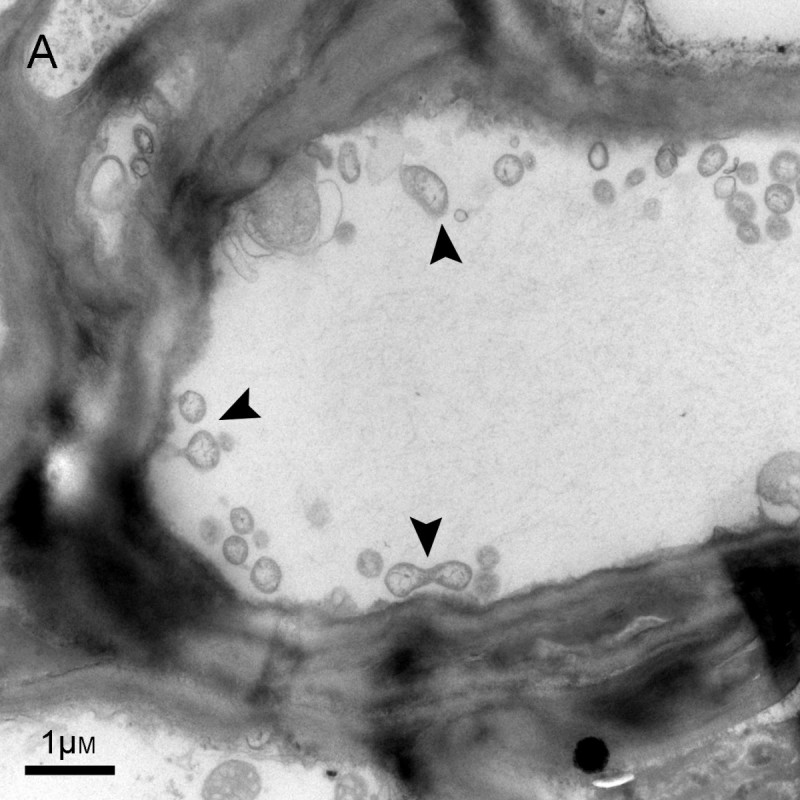
**Transmission electron micrograph of phytoplasma-like bodies (indicated by black narrow) in stem tissues of symptomatic China ixeris.** Scale bar =1 μm.

In ultra-thin sections of fasciated stems from symptomatic samples, many phytoplasma-like bodies (PLBs) were identified based on the spherical and dumbbell-shaped structures under TEM (Figure 2). The PLBs ranged from 330 to 700 nm in diameter and contained ribosome-like bodies and a central region of fibrillar material, presumed to contain DNA.

From all 30 total DNA of symptomatic samples and positive controls, PCR products of 0.8 kb (partial *tuf* gene), 1.8 kb and 1.2 kb (partial 16S rRNA gene) were generated, but no PCR product was obtained from the asymptomatic samples.

The sequences of 1,840 bp generated from symptomatic samples were identical between each other and deposited under GenBank under accession no. HM990973. The obtained sequences of 842 bp (*tuf* gene), share 100% of identity and were deposited under accession no. HM990972. NCBI BLASTn program analysis indicated that the sequences of 1,840 bp and 842 bp were homogenous to sequences of group 16SrI phytoplasmas. The HM990973 shared the highest identity of 99.67% (1,834 bp/1,840 bp) with clover phyllody phytoplasma strain CPh (accession no. AF222065) and Poa stunt phytoplasma (accession no. DQ640501), and the HM990972 had the identity of 100% with clover phyllody phytoplasma strain KV (accession no. L46369) and Wheat blue dwarf phytoplasma (accession no. DQ507200).

**Figure 3:**
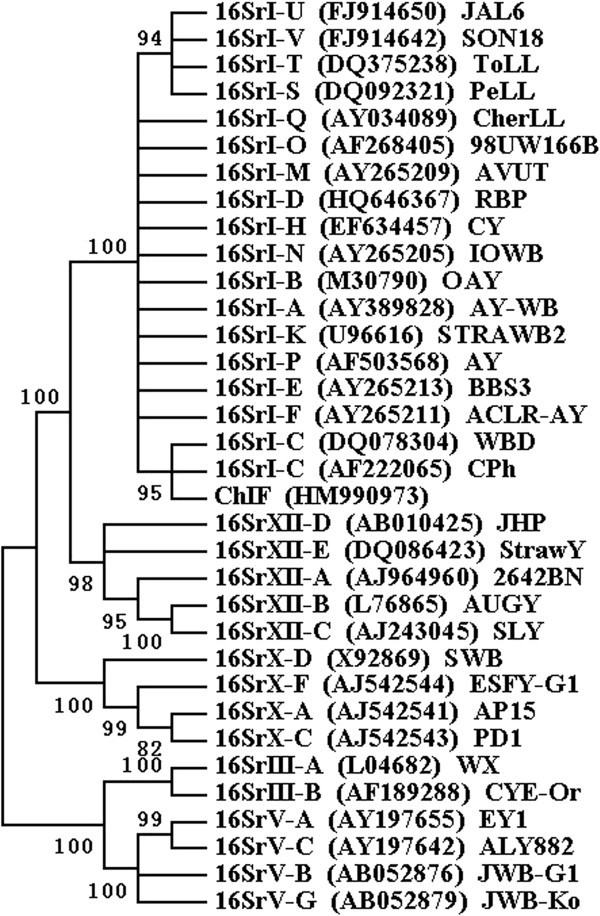
**Phylogenetic tree based on 16S rRNA gene sequences of the ChIF phytoplasma and 33 related phytoplasmas.** Numbers on the branches are confidence values obtained for 1000 replicates.

**Figure 4:**
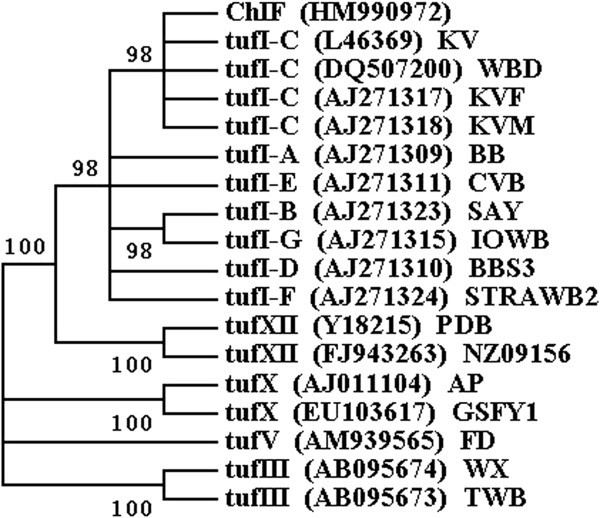
**Phylogenetic tree based on** ***tuf*** **gene sequences of ChIF phytoplasma and related 17 phytoplasmas, constructed by the maximum parsimony method.** Numbers on the branches are confidence values obtained for 1000 replicates.

As indicated by the phylogenetic trees inferred from 16S rRNA genes (Figure 3) and *tuf* genes (Figure 4), the ChIFp and phytoplasmas of group 16SrI clustered together.

**Figure 5:**
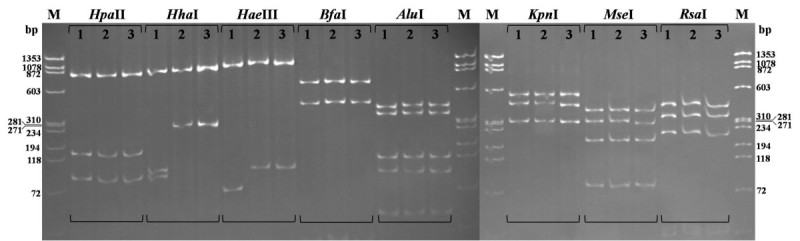
**RFLP pattern of 16S rRNA gene digested with eight enzymes** ***Hpa*** **II,** ***Hha*** **I,** ***Hae*** **III,** ***Bfa*** **I,** ***Alu*** **I,** ***Kpn*** **I,** ***Mse*** **I and** ***Rsa*** **I.** Lane M: ΦX174 DNA ladder. 1, chinaberry witches’-broom. 2, China Ixeris fasciation. 3, wheat blue dwarf.

ChIFp yielded the same RFLP pattern with WBDp, but differ from CWBp in *Hae* III and *Hha* I sites (Figure 5). After digested with enzyme *Hha* I, ChIFp and WBDp generated two bands of 872 and 310 bp, while CWBp got three bands of 872, 118 and 72 bp. And both ChIFp and WBDp yielded the bands of 1,078 and 118 bp after digestion with enzyme *Hae* III, whereas the CWBp got bands of 1,078 and 72 bp. The results suggest close correlation of ChIp and WBDp with phytoplasmas in subgroup 16SrI-C although no identical to published reference strains.

## Discussion

Based on TEM examination, PCR assays, sequencing, phylogeny and RFLP, phytoplasmas related to 16SrI-C subgroup were detected as associated with fasciation in China ixeris. There are other reports of similar association such as fasciation in lilies (*Lilium martagon*; *Liliaceae*) (Bertaccini et al., 2005; Poncarová‒Vorácková et al., 1998), *Asparagus officinalis* (*Liliaceae*) (Fránová and Petrzik, 2010), *Crotalaria spectabilis* Roth. (*Fabaceae*) (Kumar et al., 2010), Sunshine tree (*Senna surattensis* Burm. *Caesapiniaceae*) (Wu et al., 2012) and Puna chicory (*Cichrium intybus* L.) (Li et al., 2012). All the cases suggested that fasciation is a significant symptom for phytoplasma disease.

Phytoplasmas related to 16SrI-C subgroup were found in wheat (Wu et al., 2010) and so far, only two other host plants have been reported to host this kind of pathogen in China: peach trees (Zhang et al., 2013) and China ixeris and all reported in Shaanxi Province. Sequences analysis indicated that the identities of 16S rRNA genes of ChIFp (accession no. HM990973) and WBDp (accession no. DQ078304) [or peach red leaf phytoplasma (PRLp) (accession no. JX481781)] were 99.84% (1,244 bp/1,246 bp) [or 99.60% (1,241 bp/1,246 bp)], and the *tuf* genes of ChIFp (accession no. HM990972) and WBDp (accession no. DQ507200) were identical to each other. China ixeris is widely distributed as a volunteer plant and always grew in or around cultivated fields like wheat field. So it can be a significant alternative host plant for this phytoplasma. China ixeris is also a new host plant for phytoplasmas.

## Conclusion

China ixeris fasciation was confirmed associated with phytoplasmas based on results of TEM observation and PCR testing. Analyzing with the sequences of 16S rRNA gene and tuf gene of the phytoplasma suggested the phytoplasma belongs to group 16SrI, After digesting with eight enzymes, the fragments of 16S rRNA gene of the phytoplasma yielded special patterns which were consistent with the patterns for phytoplasmas of subgroup 16SrI-C in eight enzymes *Hpa* II, *Hha* I, *Hae* III, *Bfa* I, *Kpn* I and *Rsa* I sites, but different in *Alu* I and *Mse* I sites. The restriction fragment length polymorphism (RFLP) analysis supported that the associated phytoplasma was classified as related to 16SrI-C subgroup. This was the first record of phytoplasmas in China ixeris.

## Authors’ original submitted files for images

Below are the links to the authors’ original submitted files for images. Authors’ original file for figure 1 Authors’ original file for figure 2 Authors’ original file for figure 3 Authors’ original file for figure 4 Authors’ original file for figure 5 Authors’ original file for figure 6

## Abbreviations

rRNA: Ribosomal RNA
tuf: Elongation factor TU
CTAB: Cetyltrimethyl ammonium bromide
WBDp: Wheat blue dwarf phytoplasma
CWBp: Chinaberry witches’ broom phytoplasma
ChIF: China ixeris fasciation
UV: Ultra violet
TEM: Transmission electron microscope.
