# Supplementary figures and images for: Detection and identification of the phytoplasma associated with China ixeris (Ixeridium chinense) fasciation
Source: Bot Stud. 2013 Oct 31;54:52. doi: 10.1186/1999-3110-54-52 (PMC5430337; doi:10.1186/1999-3110-54-52)

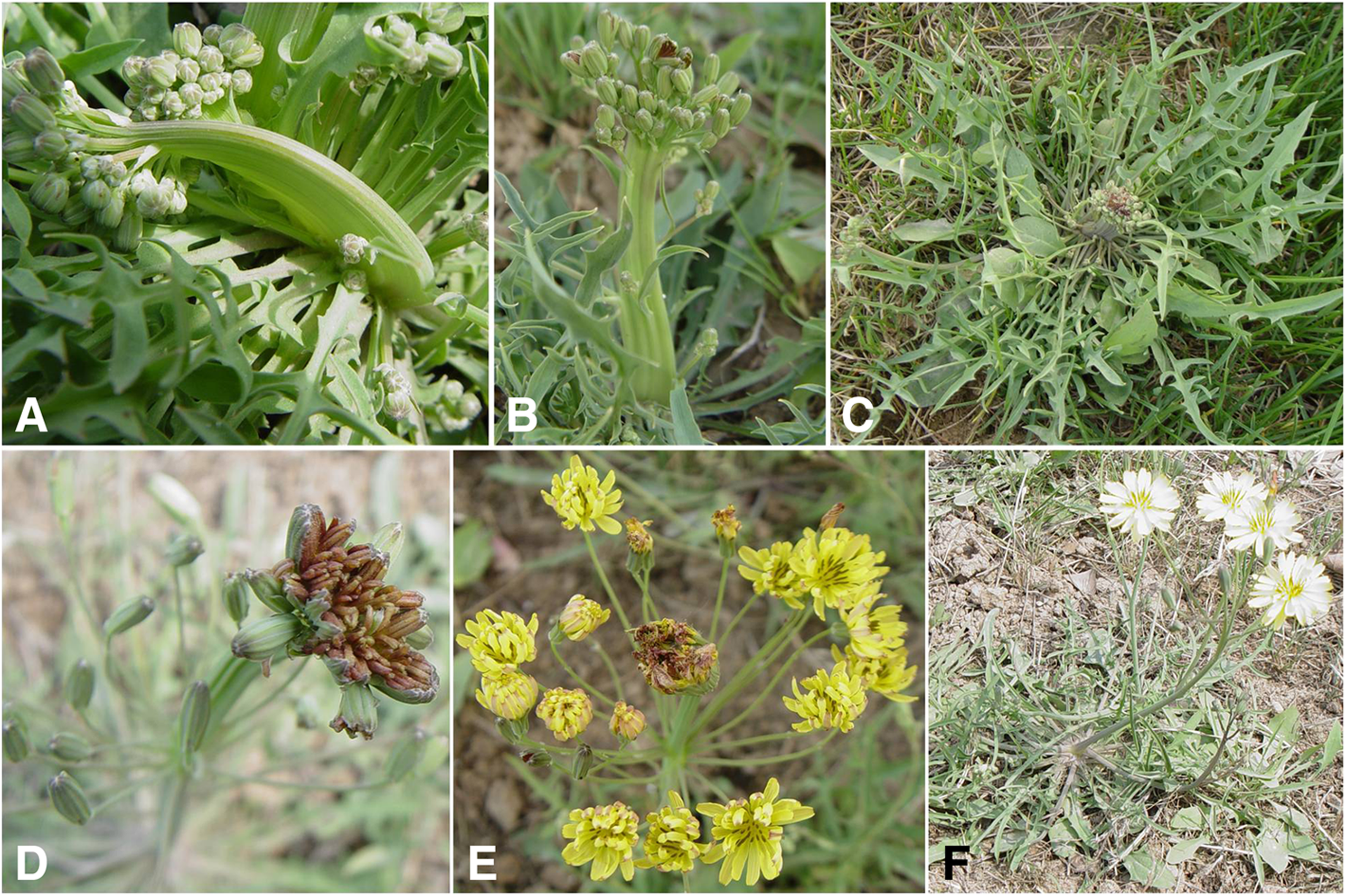

Supplement: Supplementary file 1 — Authors’ original file for figure 1 [file 40529_2013_99_MOESM1_ESM.tif]

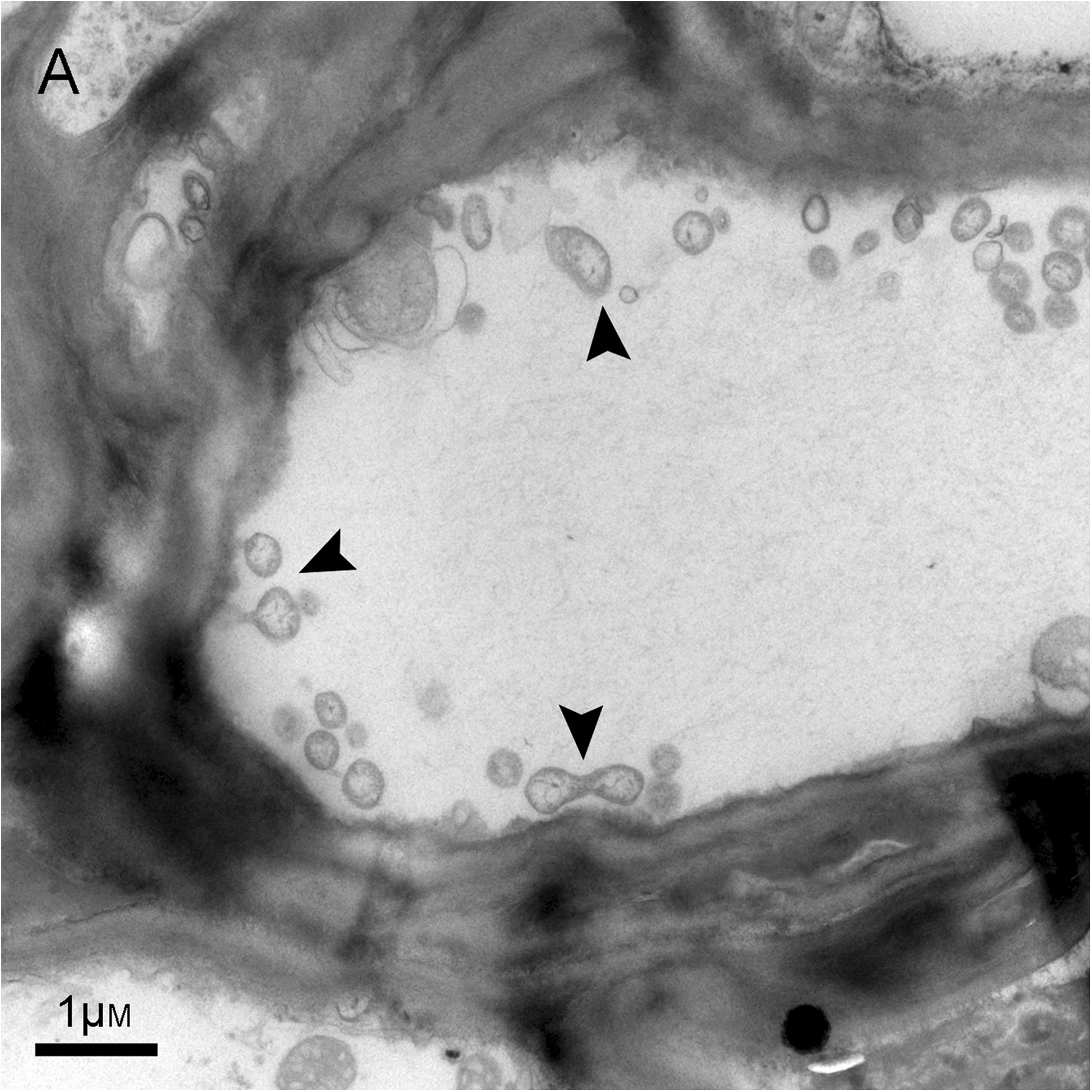

Supplement: Supplementary file 2 — Authors’ original file for figure 2 [file 40529_2013_99_MOESM2_ESM.tif]

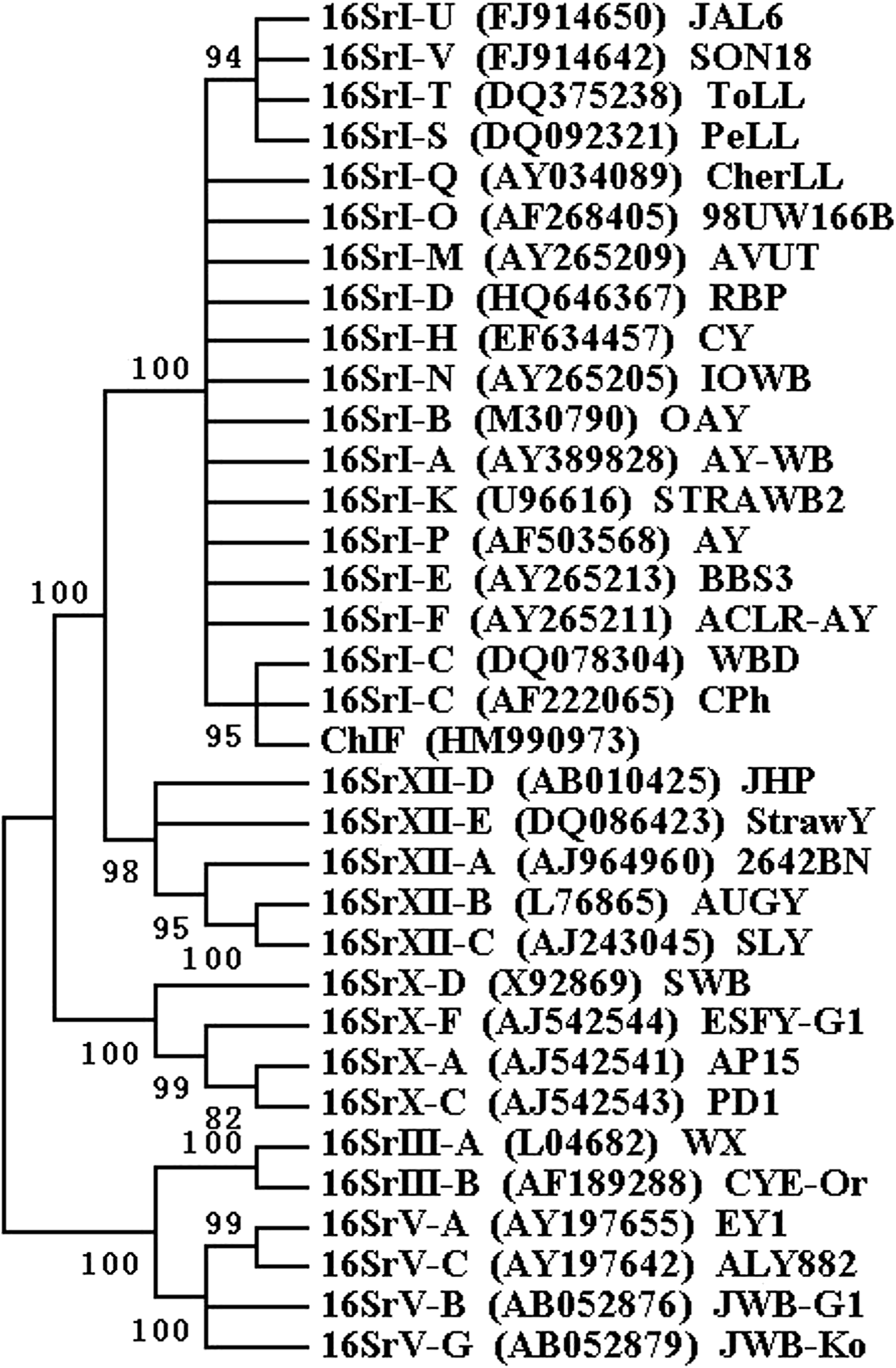

Supplement: Supplementary file 3 — Authors’ original file for figure 3 [file 40529_2013_99_MOESM3_ESM.tiff]

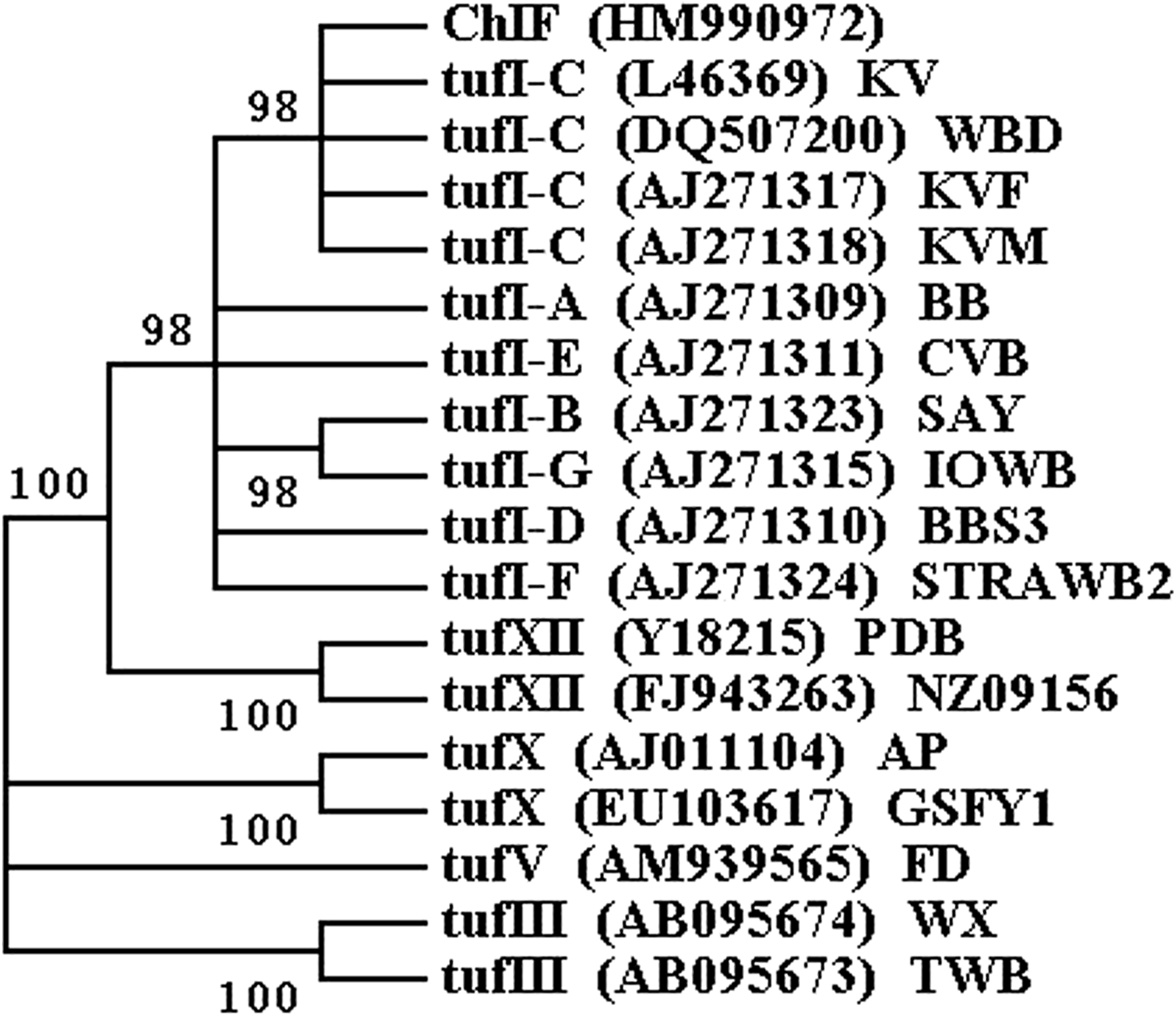

Supplement: Supplementary file 4 — Authors’ original file for figure 4 [file 40529_2013_99_MOESM4_ESM.tiff]

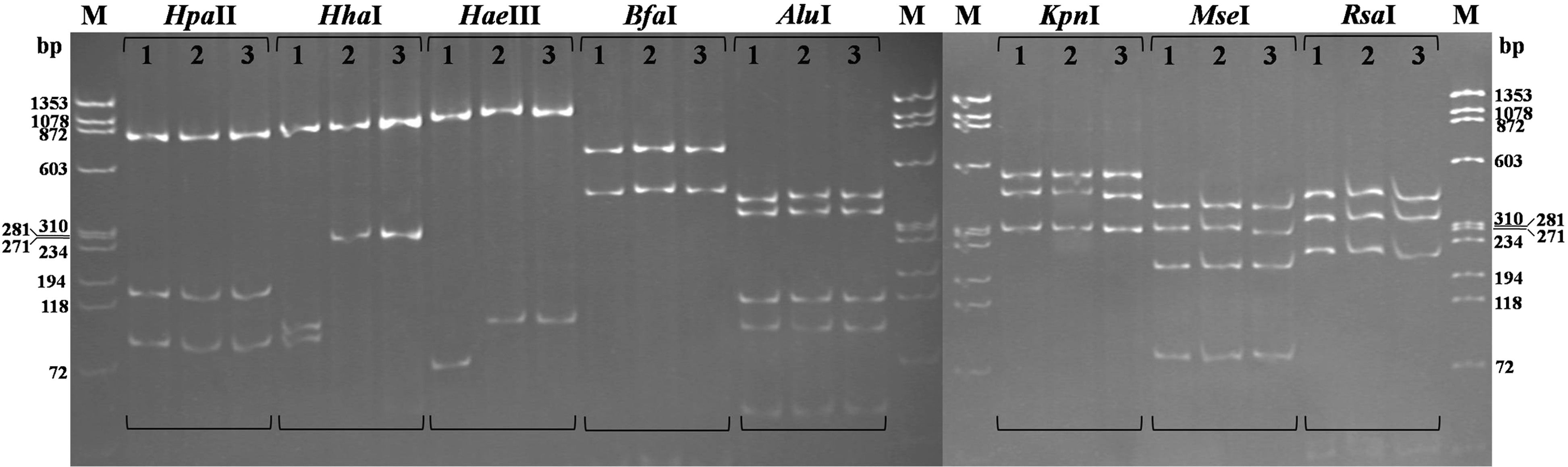

Supplement: Supplementary file 5 — Authors’ original file for figure 5 [file 40529_2013_99_MOESM5_ESM.tiff]

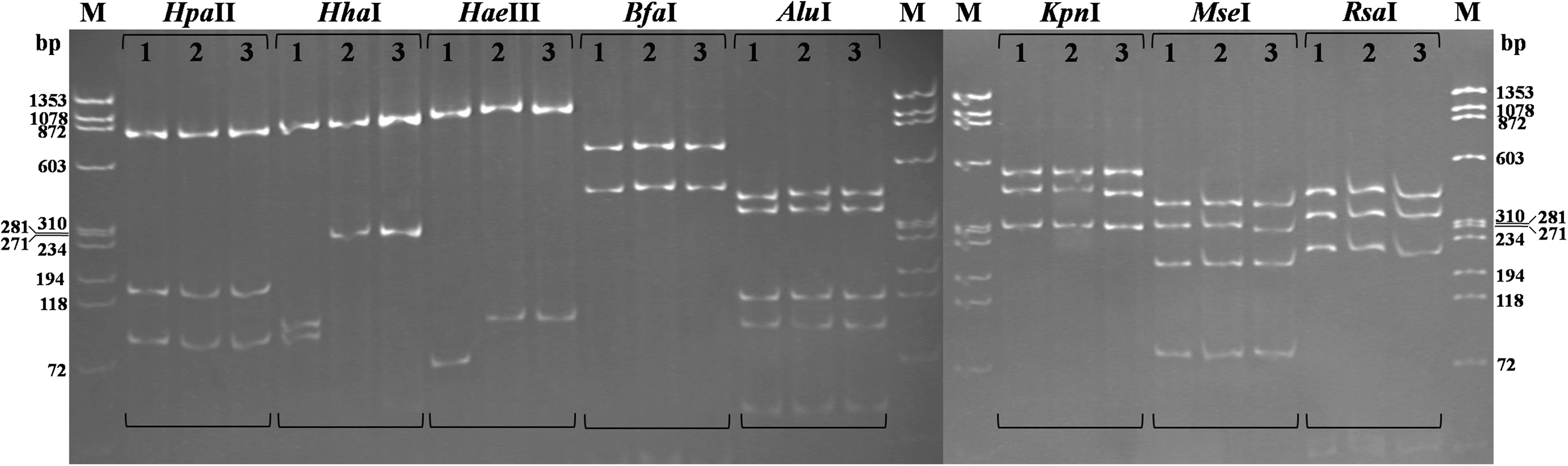

Supplement: Supplementary file 6 — Authors’ original file for figure 6 [file 40529_2013_99_MOESM6_ESM.tif]
